# Constrictive Pericarditis and Rheumatoid Nodules with Severe Aortic Incompetence

**DOI:** 10.1155/2014/524643

**Published:** 2014-02-23

**Authors:** Rory Beattie, Karen Booth, Brian Herron, Mary N. Sheppard, Haralambos Parissis

**Affiliations:** ^1^Cardiac Surgical Unit, Royal Victoria Hospital, 274 Grosvenor Road, Belfast BT12 6BA, UK; ^2^Department of Pathology, Royal Victoria Hospital, 274 Grosvenor Road, Belfast BT12 6BA, UK; ^3^Department of Histopathology, Royal Brompton Hospital, Sydney Street, London SW3 6NP, UK

## Abstract

The case of a female patient presenting with constrictive rheumatoid pericarditis and aortic incompetence secondary to valvular rheumatoid nodules is described along with a review of the literature with the aim to highlight this rare cause of aortic insufficiency.

## 1. Introduction

Baggenstoss and Rosenberg first described cardiac nodules in patients with rheumatoid arthritis (RA) in 1941, noting that these nodules had a similar histological appearance to rheumatoid nodules (RN) [1]. Subsequently there have been numerous studies describing RN in cardiac valves at postmortem but to date only a few patients have been reported to have RN on surgical excision of the aortic valve causing severe aortic incompetence.

## 2. Case Report

A 34-year-old nonsmoking Caucasian woman presented to clinic with a one-year history of worsening shortness of breath and an exercise tolerance limited to 200 metres. Past medical history included a 10-year history of seropositive RA with marked deformities in her hands. The patient also had a pericardial effusion which was drained 3 years previously and a small persistent left pleural effusion, both of which were due to RA involvement and necessitated an increase in her steroid dose. Her medications included 12 mg once daily of oral prednisolone and anti-TNF alpha antibody (Infliximab) infusions every six weeks. A loud diastolic murmur was audible on auscultation over the aortic area. Echocardiography and cardiac magnetic resonance imaging showed severe aortic incompetence with no evidence of constrictive pericarditis. Coronary angiogram revealed no coronary artery disease, but right heart catheterisation was not performed.

**Figure 1:**
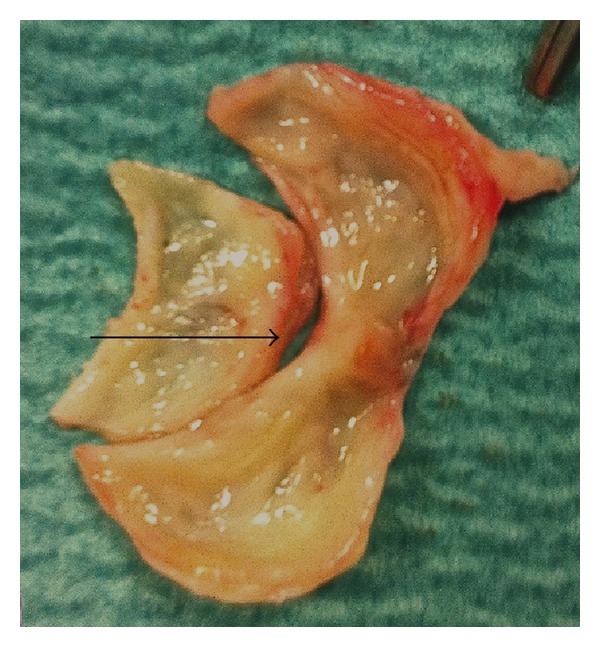
Photograph of excised thickened trileaflet aortic valve with retracted right coronary cusp (arrow) and fusion of the other two leaflets at the commissure.

**Figure 2:**
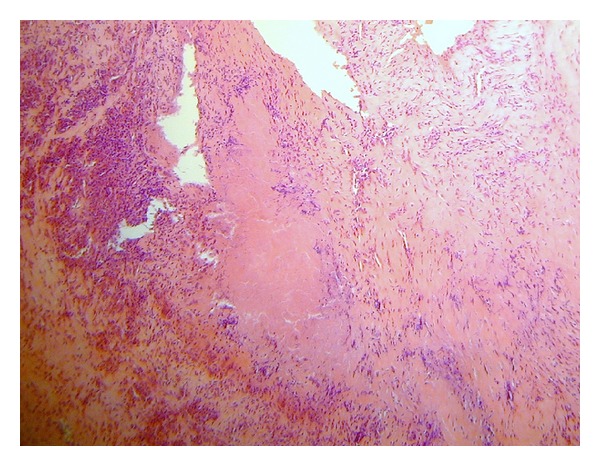
High powered microscopy of rheumatoid granulomata found in aortic valve.

The patient was admitted for elective aortic valve replacement. Sternotomy revealed a thickened 3 mm pericardium densely adherent to the epicardium and central venous pressure fell from 20 to 12 mmHg following pericardiectomy. Aortotomy was performed and a thickened trileaflet valve with a retracted right coronary leaflet and fusion of the other two leaflets at the commissure (see Figure 1) was replaced with a 21 mm biological aortic valve. Transoesophageal echocardiogram confirmed there was no paravalvular leak. At six-month review the patient's symptoms were stable, and she could walk up to half a mile and was 20-week pregnant. Histological examination of the excised valve showed predominantly myxoid degeneration and fibrosis. There were also distinct mature foci of necrosis surrounded by palisading histiocytes with an external corona of dense chronic inflammatory cells, mainly plasma cells, consistent with necrobiotic granulomas of rheumatoid arthritis (see Figure 2). There was no evidence of bacterial vegetations. The morphological features were typical of RN as seen in other sites in the body. The pericardium showed scattered chronic inflammatory cell infiltrates but no specific features of rheumatoid disease.

## 3. Discussion

Cardiac lesions in RA are classified into two categories based on histological features: those due to a generalised, nonspecific chronic inflammatory-cell infiltrate, which include perimyocarditis, valvular fibrosis, and coronary vasculitis, and those due to specific rheumatoid nodule/granulomata formation, which can arise in the peri-endo-myocardium or valves. An autopsy study of 62 patients with a diagnosis of RA found 29% had evidence of nonspecific pericarditis, 19% myocarditis, and 19% arteritis; cardiac rheumatoid nodules were found in 2 cases (3%) [2].

Subcutaneous RN is the most common extra-articular manifestation of RA, occurring in 40% of seropositive patients. Valvular abnormalities may occur in patients with RA but rarely present clinically as they are usually haemodynamically insignificant [3]. It has been hypothesised that trauma may be integral in the aetiology of RN. Valvular disease can occur in the mitral, aortic, tricuspid, and pulmonary valves in decreasing order of frequency [4], mirroring the falling levels of stress to which each valve is subjected.

The pathological features of these lesions are archetypal for this entity with the maturation of the nodule occurring in 3 stages: the acute inflammatory stage, the granulomatous stage, and the mature necrotic stage. The mature necrotic nodule has a 3-layer structure with the innermost zone containing eosinophilic acellular debris such as collagen, fibrin, and proteins. This area is surrounded by elongated palisading histiocytes and occasional multinucleated giant cells and finally by an outer layer of vascular connective tissue, macrophages, T-lymphocytes, and plasma cells.

Nonspecific inflammation and fibrosis occur in the deep layer of the valve and the valve ring causing thickening and calcification of the valve. Valvular insufficiency occurs either as a result of dilatation of the valve ring or due to contraction and destruction of the valve leaflets. RN develops in either the annulus or the central portion of the valve cusps, impeding valve function and leading to simultaneous stenosis and incompetence. The development of RN is associated with higher levels of rheumatoid 4 factor, positive anticyclic citrullinated peptide antibody titres and more severe disease.

Rare cases of stroke secondary to embolism from a ruptured valvular RN have been reported [5]. Surgical excision of the aortic valve due to severe aortic incompetence was first described in 1971 [6] with only 9 published cases in the literature over the last 40 years.

Despite up to 50% of patients with RA having involvement of the pericardium on postmortem examination, pericardial involvement does not seem to be related to disease duration or severity and less than 2% of patients exhibit symptoms [7]. A case series of 5 patients undergoing pericardiectomy for RA constrictive pericarditis found no recurrence on followup of up to 17 years [8].

In conclusion, although valvular disease in RA is a known clinical entity, very rarely it is clinically significant. We described a case of severe aortic incompetence due to a very rare pathophysiology.
